# Application of Rough Ant Colony Algorithm in Adolescent Psychology

**DOI:** 10.1155/2021/6636150

**Published:** 2021-01-14

**Authors:** Tao Cong, Lin Jiang, Qihang Sun, Yang Li

**Affiliations:** ^1^Ludong University, Yantai 264000, China; ^2^University of Toyama, Toyama 930-8555, Japan; ^3^Shanghai University, Shanghai 200444, China

## Abstract

With the rapid development of big data, big data research in the security protection industry has been increasingly regarded as a hot spot. This article mainly aims at solving the problem of predicting the tendency of juvenile delinquency based on the experimental data of juvenile blindly following psychological crime. To solve this problem, this paper proposes a rough ant colony classification algorithm, referred to as RoughAC, which first uses the concept of upper and lower approximate sets in rough sets to determine the degree of membership. In addition, in the ant colony algorithm, we use the membership value to update the pheromone. Experiments show that the algorithm can not only solve the premature convergence problem caused by stagnation near the local optimal solution but also solve the continuous domain and combinatorial optimization problems and achieve better classification results. Moreover, the algorithm has a good effect on predicting classification and can provide guidance for predicting the tendency of juvenile delinquency.

## 1. Introduction

At present, many researchers simulate biological group behavior to solve calculation problem and they have formed a theoretical system that is focused on group intelligence. Through the observation and study of biological groups, group intelligence which is produced by the cooperation and competition of individuals in biological groups can provide efficient solutions to specific problems [1]. The ant colony algorithm [2] was first proposed by Marco Dorigo et al. in 1991. They found that the ant colony can quickly find the goal by secreting a biological hormone called pheromone when the ant searches for food. Based on the above, they proposed the ant colony algorithm that is based on the positive feedback principle. According to the observation of insect scientists, although ants are visually underdeveloped, they can find the shortest path from food source to lair without any hints. After the surrounding environment changes, they adaptively search for the new best path. And, when ants are looking for food sources, they can release a hormone, called pheromone, in the path they traveled; thus, other ants within a certain range can detect. When more and more ants pass through some paths, pheromone is also more and more, so that the ants have a higher probability of choosing this path. Then repeatedly, the pheromone in this path becomes more. This selection process is called the autocatalytic behavior of ants. For a single ant, it does not have to find the shortest path, it should just choose the shortest path according to probability. However, for the whole ant colony system, they can achieve the objective effect of the original algorithm to find the optimal path. That is group intelligence [3].

Combining the rough set theory, in this paper, we propose an ant colony algorithm based on rough set, called RoughAC algorithm. It obtains a membership value through the rough set theory firstly. Then, it uses membership value to update pheromones. Exhaustive experiments show that the RoughAC algorithm can not only overcome the shortcomings of original algorithm but also improve the accuracy of classification and reduce the cost of time.

## 2. Related Work

The ant colony algorithm is inspired by the mechanism of biological evolution. By simulating the behavior of natural ant's search path, a new type of simulated evolutionary algorithm [4] is proposed, which is a main algorithm in the field of group intelligence theoretical research [5]. This method is used to solve TSP problems, allocation problems, and job-shop scheduling problems, which achieves good experimental results. Although the study time is limited, the current research shows that the ant colony algorithm has certain advantages in solving complex optimization problems [6–8], indicating that it is a promising algorithm. Ant colony algorithm is different from most application optimization algorithm based on gradient [9–11]; swarm intelligence relies on probabilistic search algorithm [12–14]. Compared with gradient methods and traditional evolutionary algorithms, the probabilistic search algorithm usually uses more evaluation functions, which has significant advantages. The advantages mainly are embodied in the absence of centralized control constraints and will not affect the entire problem due to individual failures. The solution ensures that the system is more robust. The indirect information exchange method ensures the system's scalability and parallel distributed algorithm model, making full use of multiple processors.

In the 90s, the ant colony optimization algorithm-ant system was first proposed and applied to solve the classical traveling salesman problem (TSP) in computer algorithms [15]. Starting from the ant system, the basic ant colony algorithm has been continuously developed and perfected and has been further verified in the TSP and many practical optimization problems. One of the common features of these improved versions of the Ant System (AS) is to enhance the ability to explore the optimal solution during the ant search process. The only difference between them is the search control strategy. Moreover, Ant Colony Optimization (ACO) with best results is achieved by introducing a local search algorithm [16–18]. Actually, it is a combination of the standard local search algorithm and the hybrid probabilistic search algorithm, which is conducive to improving the solution quality of the ant colony systems in the optimization problem.

Initially, there are three versions of AS: Ant-density, Ant-quantity and Ant-cycle. In Ant-density and Ant-quantity, ants update pheromone after each movement between two positional nodes. Different from Ant-density and Ant-quantity, in Ant-cycle, pheromone is updated after all ants have completed their own journey. The pheromone released by each ant is expressed as a function that reflects the quality of the corresponding trip [19]. Compared with other more general heuristic algorithms, the solving ability of these three basic algorithms is ideal in a TSP no larger than 75 cities, but the problem solving ability of AS is greatly reduced when the problem scale is extended. Therefore, the subsequent ACO research work mainly focused on the improvement of AS performance. One of the earlier improvements is the elite strategy. The idea is to give additional enhancements to all the best paths found since the algorithm was started and record the subsequent itinerary as the global optimal itinerary. When updating pheromone, these trips are weighted and the ants which pass through these trips are marked as “elites,” which increases the chances for a better trip. This improved algorithm can get a better solution faster. However, if too many elites are selected, the algorithm will lead to premature stagnation of the search due to earlier convergence to the local suboptimal solution.

The German scholar Thomas Stutzle et al. proposed another general improved ant colony algorithm: Maximum Minimum Ant Colony System (MMAS), which uses the upper and lower bounds of the given information volume, to make the quantity of pheromone on the path less than the lower limit and not exceed the upper limit, avoiding the drawback that all ants choose the same path. Gambardella et al. proposed a modified ant colony algorithm (ACA), which updates the global pheromone, solves the problem of slow convergence, and difficultly produces effective solution when solving large-scale problems. However, there are still some deficiencies in ant colony algorithm: (1) the problem of premature and early convergence is caused by the stagnation around the domain of some local optimal solutions; (2) the correct solution cannot be obtained on some combinatorial optimization problems.

Inspired by the above discussion, this paper proposes an ant colony algorithm based on rough set. The algorithm first uses the concepts of rough and upper approximation sets to obtain the membership function. Then, the membership value is used to improve the pheromone renewal of the ant colony algorithm. It can not only get the correct results in the combinatorial optimization problem but also solve the problems of stagnation, early maturity, early convergence, and so on.

## 3. Preliminary Knowledge

### 3.1. Principle of Standard AG Algorithm

Ant colony algorithm is a kind of bionic algorithm that simulates the path finding method of natural ants. During movement, ants can leave a substance called pheromone on the path it passes through for information transfer. Ants can sense this substance during exercise and use it as a guide to their movement direction. Therefore, the ant colony behavior consisting of a large number of ants shows a positive feedback phenomenon: the more ants that pass through a path, the greater the probability that the latecomers will choose the path. The algorithm is based on the following basic assumptions.

**Assumption 1 .**

Ants communicate through pheromones and the environment. Each ant responds only to the local environment around it and only affects its surrounding local environment.

**Assumption 2 .**

The ant's response to the environment is determined by its internal model. Because ants are genetic organisms, the behavior of ants is actually the adaptive performance of their genes, that is, ants are responsive adaptive entities.

**Assumption 3 .**

At the individual level, each ant only makes independent choices according to the environment; at the group level, the behavior of a single ant is random, but the ant colony can form a highly ordered group behavior through the self-organization process.

From the above assumptions and analysis, we can see that the optimization mechanism of basic ant colony algorithm contains two basic stages: adaptation stage and cooperation stage. In the adaptation phase, each candidate solution constantly adjusts its own structure according to the accumulated information. The more ants pass through the path, the greater the amount of information, the easier the path can be selected; the longer the time, the smaller the amount of information; during the collaboration phase, the candidate solutions exchange information with each other to expect a better performance solution, similar to the learning mechanism of learning automata.

The total number of ants in ants can be defined as

(1) $$\begin{array}{c} m=\overset{n}{\underset{i=1}{\sum }}b_{i}\left(t\right), \end{array}$$

where *b*_*i*_(*t*) represents the number of ants in element *i* at time *t*, *τ*_*ij*_(*t*) is the amount of information in path (*t, j*) at time *t*, and *n* represents the size of TSP.

Γ={*τ*_*ij*_(*t*)*|c*_*i*_, *c*_*j*_ ⊂ *C*} is the set of residual information on the two-by-two connections *l*_*ij*_ of elements in set C at time *t*. The amount of information on each path is equal at the initial time, and *τ*_*ij*_(0)=const is set. The basic ant colony algorithm is optimized by directional graph g=(C, L, Γ) .

The ant *k*=(*k* = 1,2, ...,m) decides its direction of movement according to the amount of information on each path during the movement. The tabu table *tabu*_*k*_(*k*=1,2,…, *m*) is used here to record the cities that the ant *k* currently walks through, and the collection is dynamically adjusted along with the *tabu*_*k*_ evolution process. During the search process, ants calculate the state transition probability based on the amount of information on each path and the heuristic information of the path. *p*_*ij*_^*k*^(*t*) represents the probability of transition of ant *k* from element *i* to element *j* at time *t*, as in fd2:

(2) $$\begin{array}{c} p_{ij}^{k}\left(t\right)=\left\{\begin{array}{c} \frac{{\left[\tau_{ij}\left(t\right)\right]}^{\alpha }\cdot {\left[\eta_{ik}\left(t\right)\right]}^{\beta }}{\sum_{s\subset {\text{allowd}}_{k}}{\left[\tau_{is}\left(t\right)\right]}^{\alpha }\cdot {\left[\eta_{is}\left(t\right)\right]}^{\beta }},\;j\in {\text{allowed}}_{k},\\ 0. \end{array}\right. \end{array}$$

where allowed_*k*_={*C* − tabu_*k*_} represents the city that ant *k* is allowed to choose next. *α* is the information heuristic factor, which reflects the role played by ants in the running process of ants during their movement. The greater the value of the ants, the more the ants tend to choose the path passed by other ants, and the stronger their collaboration. *β* is the expected heuristic factor, which reflects the importance of the heuristic information in the ant selection path during the movement of the ant. The larger the value, the closer the state transition to the greedy rule. *η*_*ij*_(*t*) is a heuristic function whose expression is as follows:

(3) $$\begin{array}{c} \eta_{ij}\left(t\right)=\frac{1}{d_{ij}}, \end{array}$$

where *d*_*ij*_ represents the distance between two adjacent cities. For ant *k*, the smaller the  *d*_*ij*_, the larger the *η*_*ij*_(*t*) and the larger the *p*_*ij*_^*k*^ .

In order to avoid the problem of flooding the heuristic information due to the excess of residual pheromone, after each ant has completed one step or completed the traversal of all *n* cities, the residual information is updated. Therefore, the amount of information on path (*i,j*) at time *t*+*n* can be adjusted according to the following formula:

(4) $$\begin{array}{c} \tau_{ij}\left(t+n\right)=\left(1-\rho \right)\cdot \tau_{ij}\left(t\right)+\Delta \tau_{ij}\left(t\right), \end{array}$$

(5) $$\begin{array}{c} \Delta \tau_{ij}\left(t\right)=\overset{m}{\underset{k=1}{\sum }}\Delta \tau_{ij}^{k}\left(t\right). \end{array}$$

where *ρ* represents the pheromone volatilization coefficient, 1 − *ρ* represents the pheromone residual factor, and Δ*τ*_*ij*_(*t*) represents the increment of pheromone on the path (*i*, *j*) in this cycle. At the initial time  Δ*τ*_*ij*_(0)=0, where Δ*τ*_*ij*_^*k*^(*t*) represents the amount of information left by the *k*-th ant on the path (*i*, *j*) in this cycle.

According to different pheromone renewal strategies, they are divided into three different basic ant colony algorithm models: Ant-Cycle model formula (6), Ant-Quantity model formula (7), and Ant-Density model formula (8). The difference is that the A method is different. In the Ant-Cycle model,

(6) $$\begin{array}{c} \Delta \tau_{ij}^{k}\left(t\right)=\left\{\begin{array}{c} \frac{Q}{L_{k}},\;\text{ if the kth ant passes }\left(i,j\right)\text{in this cycle,}\\ 0,\;else, \end{array}\right. \end{array}$$

where *Q* represents the strength of pheromone, which affects the convergence speed of the algorithm to a certain extent and *L*_*k*_ represents the total length of the path taken by the *k*-th ant in this cycle.

In the Ant-Quantity model,

(7) $$\begin{array}{c} \Delta \tau_{ij}^{k}\left(t\right)=\left\{\begin{array}{c} \frac{Q}{d_{ij}},\;\text{if the kth ant passes }\left(i,j\right)\mathrm{between}\;\left[t\;,t+1\right],\\ 0,\;\mathrm{else}\text{. } \end{array}\right. \end{array}$$

In the Ant-Density model,

(8) $$\begin{array}{c} \Delta \tau_{ij}^{k}\left(t\right)=\left\{\begin{array}{c} Q,\;\text{if the kth ant passes }\left(i,j\right)\mathrm{between}\;\left[t\;,\;t+1\right],\\ 0,\;\mathrm{else}\text{.} \end{array}\right. \end{array}$$

The algorithm contains a series of control parameters that have an important impact on the performance of the algorithm, including the following: (Algorithm 1) [20].

1. Heuristic factors *α* and *β*: *α* represents the importance of pheromone and reflects the relative importance of the amount of information remaining in the ant colony's movement in guiding the ant colony search. The greater the *α*, the greater the role of the pheromone in path selection, the greater the possibility that the ants will choose the path they have walked before, and the randomness of the search will be weakened. The smaller the *α* is, the ant colony algorithm will fall into the local optimum prematurely. When *α* *=* 0, pheromone information will not be used. Search is reduced to greedy search. *β* represents the importance of visibility and reflects the relative importance of heuristic information in guiding the ant colony search process. This heuristic information is manifested as a priori and deterministic factor in the optimization process. The greater the *β*, the greater the effect of the distance between cities on path selection, and the greater the possibility of ants choosing the local shortest path at a local point. Although the convergence speed is accelerated, the randomness is weakened and the local optimum is easy to fall into. When *β* = 0, the explicit tendency to attractive solutions is ignored, and the algorithm is equivalent to the poorly performing Simple Ant Colony Optimization (SACO). *α* and *β* determine the relative importance between the past search experience and the inherent heuristic information of the problem. They appear in most ant colony algorithms and have a crucial impact on the performance of the algorithm. Since *α* and *β* are the two major determinants of the transition probability *p*_*ij*_^*k*^ that strikes a balance between the pheromone concentration and heuristic information, the relationship between exploration and development can be handled well through the knots *a* and *β.*
2. Pheromone volatilization coefficient *ρ*: imitating the characteristics of human memory, as new information increases, old information will be gradually forgotten and weakened. Therefore, *ρ* is introduced to represent the volatilization rate of information cables. In order to prevent unlimited accumulation of information, the value range of *ρ* is set to [0,1]. The size of *ρ* is directly related to the global search ability and convergence speed of the ant colony algorithm. If *ρ* increases, pheromone volatilization speeds up, and it is less sensitive to past historical experience, highlighting the influence of the information left by the recent route seven on the choice.
3. Pheromone intensity factor *Q*: *Q* represents the total view of information released by the ants in a cycle or a process. To a certain extent, it affects the convergence speed of the local algorithm. The larger the *Q*, the more information requests are left each time the ant passes, and the faster the accumulation of information requests on the traversed path. Enhancing the positive feedback of the ant colony search is helpful to the rapid convergence of the algorithm.

### 3.2. Rough Set

Rough set theory [21–23] is a new mathematical tool for dealing with uncertainties and inaccuracies. It is very important for artificial intelligence and cognitive science, and it provides a very effective theoretical framework for information processing in the fields of machine learning, data mining, knowledge acquisition, decision analysis and support systems, pattern recognition, expert systems, granular computing, approximate reasoning, control science, and so on. At the same time, the rough set method has important applications in many aspects such as medicine, finance, meteorology, graphic processing, speech recognition, and character recognition. Therefore, since its inception, it has received extensive attention. The main problems of rough set theory processing include knowledge reduction of knowledge expression system, discovery of knowledge relevance, evaluation of data significance, acquisition of decision control algorithm, approximate classification [22, 24, 25], and approximate reasoning. Rough sets can express not only fuzzy concepts but also clear concepts. Both fuzzy set and rough set theory can deal with the problems of uncertainty and inaccuracy. They are the generalization and important development of classical set theory. However, their focus is different. For example, the degree of membership of the object *x* in the fuzzy set theory does not depend on other objects in the domain, which is generally given directly by the expert or obtained by statistical methods. It can reflect the changing laws of objective things, but it also has a strong subjectivity and lack of accuracy. The value of the membership function of the object in the rough set theory depends on the knowledge base. It can be obtained directly from the data to be processed, without any external information. Therefore, it is more objective to use it to reflect the ambiguity of knowledge. At the same time, there is also a connection between the two, because given an equivalence relationship (knowledge) *R* on the universe of *U* and *U*, any subset *A* (concept) of the universe actually corresponds to a fuzzy set *B*. Its upper and lower approximations are equivalent to the kernel and branch set of the fuzzy set, namely,

(9) $$\begin{array}{c} \underline{R}\left(A\right)=\text{Core}\left(\mu_{A}\right)=\left\{x|\mu_{A}\left(x\right)=1\right\},\\ \bar{R}\left(A\right)=\text{Suup}\left(\mu_{A}\right)=\left\{x|\mu_{A}\left(x\right)>0\right\}. \end{array}$$

From this, we can see that the lower approximation is a 1 cut set of *A*, and the upper approximation is exactly the strong 0 cut set of *A*. In short, fuzzy sets and rough sets can describe the uncertainty of knowledge, but their respective focus is different. There is a strong complementarity between fuzzy set theory and rough set theory. Two theories have been shown stronger functions through optimization and integration to deal with the uncertainty and incompleteness of knowledge. For example, projection set theory is developed in the framework of fuzzy set theory, but the method of processing information is similar to a rough set, and its application in some fields shows advantages. There are many fuzzy rough set hybrid models that solve practical problems that some single models cannot solve. This shows that the fusion of two theories is an effective way to solve complex problems.

## 4. Rough Ant Colony Algorithm

### 4.1. Related Concepts

The ant colony algorithm not only can be intelligently searched and globally optimized but also has the characteristics of robustness, positive feedback, distributed computing, and easy combination with other algorithms, constructiveness, which can be used for artificial ants to join the characteristics of natural ants such as prospective and backtracking according to the needs. The advent of ant colony algorithm provides a powerful tool for solving complex optimization problems in many fields. Although ant colony algorithm has many advantages, it also has some defects. Compared with other methods, the algorithm generally requires a long search time, and the complexity of the ant colony algorithm can reflect this. Although the increase in computer computing speed and the inherent parallelism of the ant colony algorithm can alleviate this problem to some extent, it is still a big obstacle for large-scale optimization problem. Moreover, this method is prone to stagnation phenomenon. That is, after the search reaches a certain level, the solutions found by all individuals tend to be consistent, and the solution space cannot be further searched, which is not conducive to finding a better solution. In the ant colony algorithm, ants always rely on the feedback information of other ants to strengthen the learning and do not consider the accumulation of their own experience. Such blind and obedient behaviors tend to lead to premature and stagnation phenomena, and thus the convergence speed of the algorithm becomes slower. In view of the above drawbacks, the ant colony classification algorithm using rough sets can solve the above problems better.

**Definition 1 .**

Approximate accuracy

Given an equivalence relation *R* on the universe of *U* and *U*, the approximate accuracy and roughness of the set *X* defined by the equivalence relation *R* is as follows:

(10) $$\begin{array}{c} \alpha_{R}\left(X\right)=\frac{\underline{R}\left(X\right)}{\bar{R}\left(X\right)},\\ \rho_{R}=1-\alpha_{R}\left(X\right). \end{array}$$

The inaccuracy of the set is caused by the existence of the boundary domain. The larger the boundary, the lower the accuracy. For each *R* and *X*⊆U, 0 ≤ *α*_*R*_(*X*) ≤ 1. When *α*_*R*_(*X*)=1, the *R*-boundary domain of *X* is an empty set, so the set *X* is an exact set of R; when *α*_*R*_(*X*) < 1, the set *X* has a non-null *R*-boundary domain, so the set *X* is a *R*-rough set; when *X* is an empty set, we specify *α*_*R*_(*X*)=*α*_*R*_(∅)=1. The *R*-roughness of *X* is opposite to the accuracy, which reflects the incompleteness of our understanding of the category of expression of Set *X* under knowledge *R*.

Let us give an equivalence relationship *R* between the discourse *U* and the universe *U*, and a subdivision *π*(*U*)=*X*={*X*_1_, *X*_2_, ⋯, *X*_*n*_} over the universe *U*, and this division is independent of the knowledge *R*. Among them, subset *X*_*i*_(*i*=1,2, ⋯, *n*) is an equivalence class that divides *π*(*U*). The lower and upper approximations of *R* for *π*(*U*) are

(11) $$\begin{array}{c} \underline{R}\left(\pi \left(U\right)\right)=\underline{R}\left(X_{1}\right)\cup^{}\underline{R}\left(X_{2}\right)\cup^{}\cdots \cup^{}\underline{R}\left(X_{n}\right) \end{array}$$

(12) $$\begin{array}{c} \bar{R}\left(\pi \left(U\right)\right)=\bar{R}\left(X_{1}\right)\cup^{}\bar{R}\left(X_{2}\right)\cup^{}\cdots \cup^{}\bar{R}\left(X_{n}\right) \end{array}$$

(13) $$\begin{array}{c} \alpha_{R}\left(\pi \left(U\right)\right)=\frac{\sum_{i=1}^{n}\left|\underline{R}\left(X_{i}\right)\right|}{\sum_{i=1}^{n}\left|\bar{R}\left(X_{i}\right)\right|}=\frac{car\;d\left(\underline{R}\left(\pi \left(U\right)\right)\right)}{car\;d\left(\bar{R}\left(\pi \left(U\right)\right)\right)}=\frac{\underline{R}\left(\pi \left(U\right)\right)}{\bar{R}\left(\pi \left(U\right)\right)} \end{array}$$

(14) $$\begin{array}{c} \gamma_{R}\left(\pi \left(U\right)\right)=\frac{\sum_{i=1}^{n}\left|\underline{R}\left(X_{i}\right)\right|}{\left|U\right|}=\frac{car\;d\left(\underline{R}\left(\pi \left(U\right)\right)\right)}{car\;d\left(U\right)}=\frac{\underline{R}\left(\pi \left(U\right)\right)}{\left|U\right|} \end{array}$$

Based on the above definition, the approximate *R-*classification accuracy and the approximate classification quality of the partition *π*(*U*) are, respectively, defined as follows:

**Definition 2 .**

Importance degree.

Given a knowledge base *K*=(*U*, *S*), *R* ∈ *IN*  *D*(*K*) represents a group or individual system parameters describing the characteristics of the system. ∀*X*⊆*U* and the partition *π*(*U*)=*X*={*X*_1_, *X*_2_, ⋯, *X*_*n*_} of the universe *U* that are independent of the system parameter *R*, and the importance degree of the set *X* regarding the system parameter *R* is expressed as

(15) $$\begin{array}{c} sig_{R}\left(X\right)=\frac{\left|U-bn_{R}\left(X\right)\right|}{\left|U\right|} \end{array}$$

(16) $$\begin{array}{c} sig_{R}\left(\pi \left(U\right)\right)=\frac{\sum_{i=1}^{n}U-bn_{R}\left(X_{i}\right)}{n\left|U\right|}, \end{array}$$

where *bn*_*R*_(*X*) represents the boundary domain of the *X*, the *U* representation domain. When *sig*_*R*_(*X*)=1, it is shown that *R* can accurately describe the set *X*; when *sig*_*R*_(*X*)=0, it indicates that *R* cannot determine whether the sum of any element in the universe *U* belongs to *X*.

**Definition 3 .**

Roughness.

Intuitively, the rough set theory does not require any prior knowledge about the inaccuracy of things. It only depends on the given expression system and directly calculated by the upper and lower approximation operators. This is totally different from the theory of probability and fuzzy set theory. From the perspective of rough set theory, the inaccuracy of objective things is caused by the limited nature of the knowledge we have. In other words, it is caused by the result of the limited ability of the objects to be classified. Therefore, people can deal with inaccurate numerical features through classification without any prior knowledge and then express the accuracy of the concept. The formula of roughness is shown as follows:

(17) $$\begin{array}{c} pow_{R}\left(X\right)=1-\alpha_{R}\left(X\right) \end{array}$$

**Definition 4 .**

Degree of membership.

Introducing the membership value obtained by the approximate accuracy of the rough set can eliminate the stagnation phenomenon of the basic AG algorithm near the neighborhood of one or some local optimal solutions, because the approximate accuracy itself has a balance between maintaining global and local search capabilities. This makes each search ant leave an appropriate amount of pheromone on the path that may be the optimal solution component based on the investigation table. The formula of membership degree is

(18) $$\begin{array}{c} P=\left\{\begin{array}{c} pow_{min}-\frac{\left(pow_{max}-pow_{min}\right)*\left(f-f_{min}\right)}{f_{avg}-f_{min}}f\leq f_{avg}\\ pow_{max}f>f_{avg}, \end{array}\right. \end{array}$$

where *pow*_*max*_ and *pow*_*min*_ are the maximum and minimum membership values, *f* is the current information amount; *f*_*min*_ is the minimum information amount of the current ant colony; and *f*_*avg*_ is the average value of the minimum information amount of the current ant colony.

Rough ant colony algorithm uses the above definition to modify the amount of information on the path and gets the following expression:

(19) $$\begin{array}{c} \tau_{ij}\left(t+n\right)=\left(1-\rho \right)\cdot \tau_{ij}\left(t\right)+\Delta \tau_{ij}+P\cdot \tau_{ij}\left(t\right). \end{array}$$

### 4.2. Algorithmic Description

According to the above definition and analysis, the steps to improve the algorithm are as follows (Algorithm 2) [20].

The most critical step of this algorithm is the update pheromone concentration of Step 7. When all ants reach the end point, the information concentration of each path must be updated again. Therefore, the update of the pheromone is divided into two steps: (1) after each round, the pheromone on all paths in the problem space will evaporate, and a membership function is obtained by the rough set theory. (2) All ants release the pheromone on the edge of their own turn according to the length of their own path and then use equation (19) to update the pheromone.

## 5. Experimental Results and Analysis

### 5.1. Experiments in TSP Instances

In order to test the results of RoughAC algorithm's optimal path, this paper applies ant colony algorithm to TSP and implements it with Matlab. The test algorithm example is selected from TSPLIB. The number in the case name indicates the number of cities. In this algorithm, *α*=1 and *β*=2. The MMAS is compared with the algorithm of this paper to test the optimization effect of RoughAC algorithm. MMAS is the largest and smallest ant system that uses a local update strategy and a global update strategy.  Table 1 is the experimental data.

**Figure 1:**
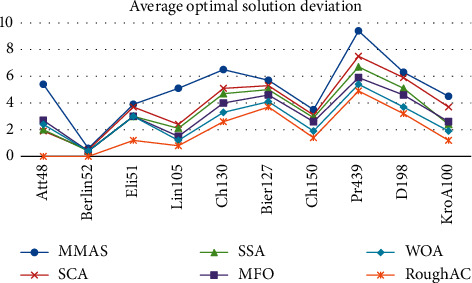
Comparison of average optimal solution deviation.

We know that, in solving small-scale, medium-scale, or large-scale TSP problems, the superiority of the algorithm can be seen by comparing the quality of the solution. Here, the MMAS, SCA, SSA, MFO, and WOA algorithms are compared with the improved RoughAC algorithm. Through data statistics, it can be understood that the performance of the improved algorithm is excellent.  Table 2 shows the statistics of 10 experiments on the experimental data of Table 1:

The comparison of the deviation of the average optimal solution of the algorithm is shown in Figure 1.

Among them, the TSP instance is the corresponding TSP problem, and the optimal solution of the algorithm represents the best one that obtained in 10 runs. The algorithm's average optimal solution represents the average of the 10 best paths. The first deviation represents the deviation of the conventional ant colony algorithm from the improved ant colony algorithm. The second deviation represents the deviation between the best one and the best known solution found by the two algorithms. From the first deviation, we can see that in the column of the optimal value deviation, the deviation of all the problems is positive and the average deviation of the MMAS algorithm is 2.9% less than that of the RoughAC, which not only shows that the quality of the improved algorithm is higher than MMAS in all the problems, but the gap is also very large. In terms of the average deviation, the deviation of all the problems is positive, and the average deviation of the MMAS is 3.19%. This shows that the improved algorithm is far more stable than MMAS in the stability of the solution. In the second deviation, it can be seen that RoughAC has some deviations of 0 and has a very high solution quality. Therefore, RoughAC algorithm is better than MMAS algorithm both in solving quality and solving stability.

### 5.2. Experiments in Data Classification

In order to test the result of the RoughAC classification algorithm, experiments were performed using 13 true classification datasets on UCI. Compared with the MMAS classification algorithm, the ACA algorithm, the KNN algorithm, and SVM algorithm, MMAS is an improved ant colony algorithm and ACA is an original ant colony classification algorithm. These types of algorithms are representative ant colony classification algorithms. The actual dataset on the experimental UCI is shown in Table 3. These datasets are commonly used test datasets and have a certain degree of differentiation in the number of attributes and the number of clusters. For the case where the attributes of some datasets are not in the same order of magnitude, they are normalized.

The evaluation criteria for the experimental results were accuracy (*Acc*) and time. Accuracy is widely used in the field of information retrieval and statistics to evaluate the quality of classification results. The time is used to represent the speed of the classification algorithm and to reflect the time complexity of the algorithm. The larger the index value of the accuracy rate, the better the classification effect. The smaller the time, the higher the efficiency of the representative algorithm. The definition of accuracy is as follows:

(20) $$\begin{array}{c} \text{Acc}=\frac{\sum_{i=1}^{k}a_{i}}{\left|U\right|}. \end{array}$$

Among them, *k* is the number of clusters, *a*_*i*_ represents correctly classified into clusters, and *U* is the whole sample.

The RoughAC classification algorithm experiment uses 13 real datasets provided by UCI to train and test the algorithm. The data are shown in Table 3. The experimental method adopts a random test method. The 9/10 data in the dataset are selected randomly as training set, and the remaining 1/10 data are used to test the classification performance. For each dataset, 100 random samples were taken and the average of the 100 classification performances was counted as the result of this classification.  Table 4 shows the accuracy of each classification method.

Table 5 shows the expected convergence time for each method on each dataset.

**Figure 2:**
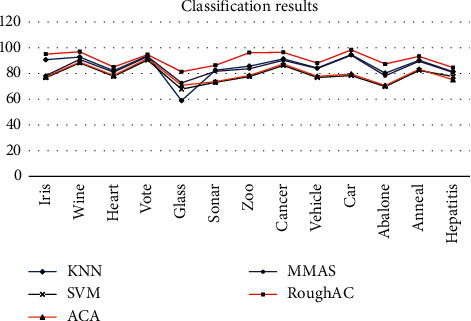
Comparison of classification results.

According to the data in Table 4 and Figure 2, it can be seen that the accuracy of the RoughAC algorithm is higher than that of the ACA algorithm and the MMAS algorithm. Therefore, it can be seen that the improved RoughAC algorithm based on rough sets can be used for classification problems, the accuracy rate is greatly improved compared with the original ant colony algorithm, and the accuracy rate of the other improved MMAS classification algorithms is also significantly improved. Therefore, RoughAC is effective for classification. The expected convergence time describes the expected time for the first time that the ant colony algorithm reaches the global optimal solution with probability. The smaller the convergence time is, the faster the ant colony algorithm converges and the higher the efficiency is. This algorithm uses 16 datasets to test the expected convergence time of the algorithm and other improved ant colony algorithms. It can be seen from Table 5 that the expected convergence time of RoughAC algorithm on all test datasets is smaller than that of ACA and MMAS. Therefore, the convergence speed of this algorithm is faster than that of the other two algorithms, and its efficiency is also higher than that of the other two algorithms.

### 5.3. Experiments on Adolescents' Blind Obedience Psychology and Criminal Behavior

In order to prove the validity of the correlation prediction model between adolescent blind obedience and criminal behavior based on multiobjective evolutionary algorithm, an experiment is needed. The experimental data were collected from 12 prisons (including women's prisons) in 8 provinces, autonomous regions and municipalities directly under the Central Government. The number of prisoners was 1000, including 500 males and 500 females. The average age was under 18 years old. The correlation between juvenile blind obedience psychology and criminal behavior is predicted by using the algorithm and the original algorithm, respectively. Under different experimental times, the comprehensive performance of the correlation prediction of different models is compared. The accuracy, efficiency, and stability of different models are taken as the evaluation indexes of the comprehensive performance. The result is shown in the figure.

**Figure 3:**
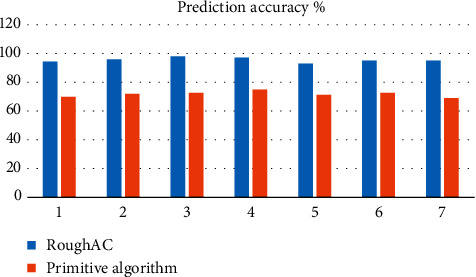
Comparisons of prediction accuracy of different algorithms.

**Figure 4:**
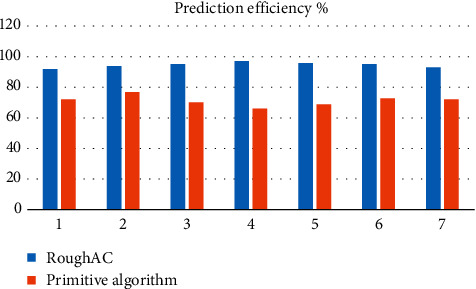
Comparison of prediction efficiency of different algorithms.

**Figure 5:**
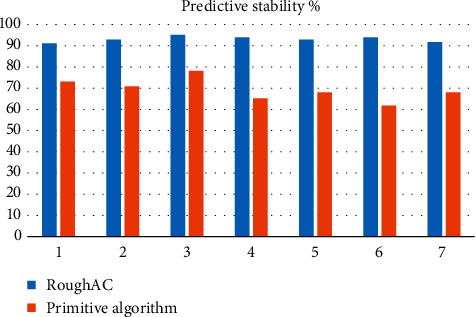
Predictive stability comparison of different algorithms.

Analysis of Figure 3 shows that the average prediction accuracy of this model is about 93.94%, the average prediction accuracy of traditional model is about 70.72%, and the prediction accuracy of this model is about 32.81% higher than that of traditional model. Analysis of Figure 4 shows that the average prediction efficiency of this model is about 93.71%, the average prediction efficiency of traditional model is about 71.02%, and the prediction efficiency of this model is about 31.93% higher than that of traditional model.  Figure 5 shows that the average predictive stability of this model is about 92.72% and that of the traditional model is about 70.19%. The predictive stability of this model is about 32.08% higher than that of the traditional model.

**Figure 6:**
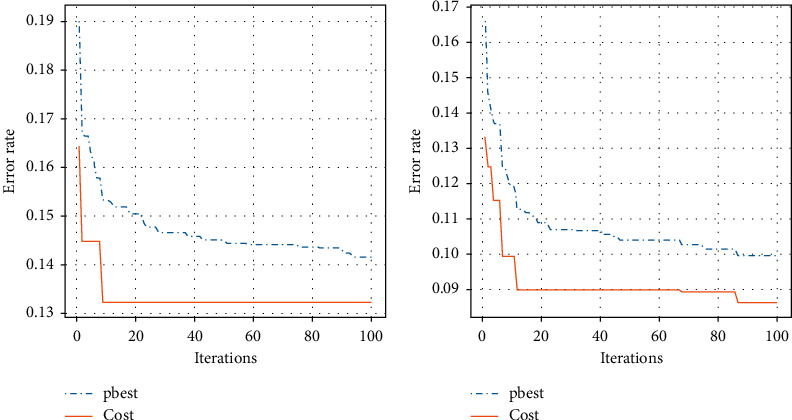
Error rate in the different iteration.

Figure 6 shows the error rate that changes over different iterations. The lower the error rate, the accuracy of the model is higher. As the number of iterations increases, the error rate is correspondingly lower and lower. The solid line represents the cost. As the number of iterations increases, the error rate of the cost also decreases. The more the iterations, the penalty cost assigned to the model is more accurate and the accuracy of model is higher. The dotted line represents the error rate of the current optimal value of the particle. It also decreases as the number of iterations increases, indicating that the current optimal value of the particle is also constantly approaching the global optimal value, and the accuracy of the model is higher.

Figure 5 shows the accuracy of the model under different feature subsets. It can be seen from the figure that all features are not required to achieve high accuracy, and only half or less of them can be used to achieve satisfactory accuracy, which reduces the running time and reduces the required memory space. The size of the feature subset will affect the accuracy of the model to a certain extent. It can be seen from the figure that the accuracy is relatively high when the number of feature subsets is substantially half of the overall feature subset. That is to say, a complete feature set is not required to achieve satisfactory accuracy for a dataset with a large dimension. It is only necessary to be able to take half or less of the feature set without reducing the accuracy of the model.

**Figure 7:**
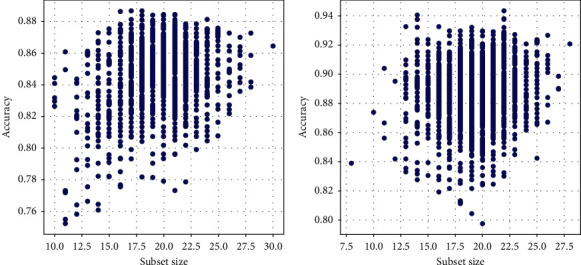
Accuracy of different subset sizes.

It can be seen from Figure 7 that the feature subset of the RoughAC model is more accurate and the number of feature subsets used is also less, so that the model has less running time and memory. On the contrary, when the number of feature subsets reaches the maximum, the accuracy of the model is reduced, so that it is known that there is a higher accuracy without using more features, and some features may affect the accuracy of the model. The ant colony algorithm is less accurate, and due to using more features, the runtime and memory of the model will increase accordingly. Overall, the performance of the proposed model is better both in terms of accuracy and the number of feature subsets used.

## 6. Conclusion

This paper takes teenagers as the object and studies the tendency of juvenile delinquency as motivation and proposes a rough ant colony classification algorithm that updates the pheromone by membership value. The algorithm uses the concept of upper and lower approximate sets in rough sets to determine the degree of membership and then uses the value of the degree of membership to update the pheromone. It solves the shortcomings of the original algorithm such as continuous domain and combination optimization, premature convergence, and local optimal solution. A large number of experiments show that in terms of classification, RoughAC algorithm not only has more advantages than AGA, MMAS, and other ant colony algorithms but also has a higher accuracy rate than KNN, SVM, and other classification algorithms. This enables the RoughAC algorithm to accurately classify juvenile crimes for better management. In terms of prediction, the RoughAC algorithm can accurately predict the tendency of young people to commit crimes through existing problems. This algorithm has made a great contribution to the study of youth psychological crime.

## Figures and Tables

**Algorithm 1:**
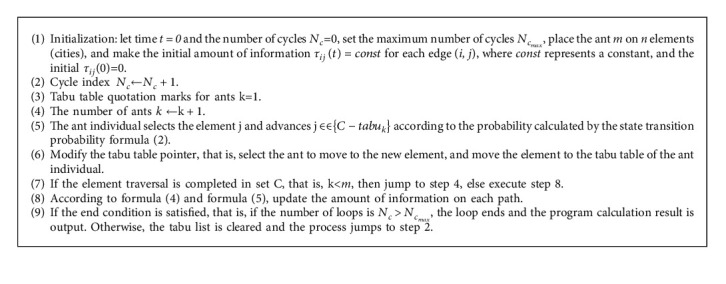
Taking TSP as an example, the specific implementation steps of the basic ant colony algorithm are as follows :

**Algorithm 2:**
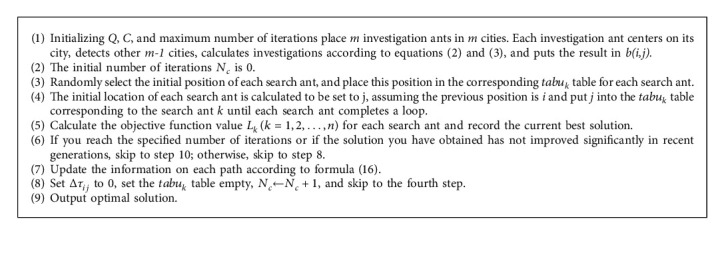
Rough ant colony algorithm flow.

**Table 1:** TSPLIB experimental data.

TSP examples	Optimal solution
Att48	33522
Berlin52	7542
Eli51	426
Lin105	14379
Ch130	6110
Bier127	118282
Ch150	6528
Pr439	107217
D198	15780
KroA100	21282

**Table 2:** Experimental optimal solution.

TSP example	Algorithm optimal solution						Deviation %					
	MMAS	SCA	SSA	MFO	WOA	RoughAC	MMAS	SCA	SSA	MFO	WOA	RoughAC
Att48	35442	32940	34182	32673	34291	33530	5.4	1.9	2.0	2.7	2.4	0
Berlin52	7592	7584	7577	7571	7579	7543	0.6	0.5	0.4	0.4	0.4	0
Eli51	442	440	441	441	441	430	3.9	3.7	3.0	3.0	3.0	1.2
Lin105	15105	15010	13892	14970	13924	14410	5.1	2.4	2.1	1.5	1.2	0.8
Ch130	6521	6482	6475	6361	6329	6269	6.5	5.1	4.7	4.0	3.3	2.6
Bier127	125050	124545	124287	123328	123295	122720	5.7	5.3	5.0	4.6	4.1	3.7
Ch150	6755	6396	6694	6427	6639	6590	3.5	3.1	2.9	2.6	1.9	1.4
Pr439	117310	116274	115742	111923	113194	112520	9.4	7.5	6.7	5.9	5.4	4.9
D198	16340	14765	16337	14972	16314	15980	6.3	5.9	5.1	4.6	3.7	3.2
KroA10	22832	22753	22337	22358	21984	21530	4.5	3.7	2.4	2.6	1.9	1.2

**Table 3:** UCI real dataset.

Dataset	Number of instances	Attribute	Class number
Iris	150	4	3
Wine	178	13	3
Heart	270	13	2
Vote	435	16	3
Glass	214	10	7
Sonar	208	60	2
Zoo	101	17	7
Cancer	699	10	2
Vehicle	846	18	4
Car	1728	6	4
Abalone	4177	8	3
Anneal	798	38	5
Hepatitis	155	19	2

**Table 4:** Comparison of classification results.

Dataset	KNN	SVM	ACA	MMAS	SCA	SSA	MFO	WOA	RoughAC
Iris	90.67	76.70	77.32	78.26	89.24	93.82	92.61	93.08	95.0
Wine	92.70	88.20	88.78	90.87	91.38	94.57	93.26	92.95	96.85
Heart	82.10	77.80	78.4	81.00	81.99	83.81	82.98	82.26	84.96
Vote	93.70	90.40	91.64	93.16	92.85	93.30	92.82	93.17	94.57
Glass	58.88	67.78	70.79	72.65	77.65	80.41	79.73	79.52	81.32
Sonar	82.56	73.10	73.56	81.67	82.88	85.48	84.26	83.97	86.32
Zoo	85.64	77.58	78.45	83.64	90.71	95.04	93.75	93.08	96.14
Cancer	91.30	86.20	87.28	90.24	91.67	95.32	94.63	94.09	96.35
Vehicle	84.24	76.82	77.79	83.86	84.93	86.85	85.71	85.14	88.01
Car	94.56	78.34	79.56	94.14	95.60	97.74	96.62	96.73	98.28
Abalone	80.23	69.78	70.64	78.15	83.85	86.63	85.55	85.08	87.35
Anneal	90.21	82.35	83.29	89.48	90.98	93.01	91.96	91.20	93.24
Hepatitis	81.40	77.60	75.12	80.75	82.48	83.71	92.84	92.16	84.58

The results of the classification are compared as shown in Figure 2.

**Table 5:** Comparison of expected convergence time.

Dataset	KNN	SVM	ACA	MMAS	SCA	SSA	MFO	WOA	RoughAC
Iris	0.56	0.49	1.24	1.02	1.08	1.24	1.33	1.17	0.19
Wine	0.75	0.62	1.25	0.60	1.05	1.56	1.48	1.41	0.54
Heart	1.45	1.23	1.17	0.65	1.53	1.72	1.81	1.68	0.60
Vote	1.94	1.72	1.14	1.02	1.85	2.19	2.07	1.98	0.89
Glass	1.89	1.77	1.03	0.75	1.97	2.36	2.17	2.02	0.54
Sonar	2.78	2.10	1.2	0.81	2.80	3.86	3.14	2.98	0.73
Zoo	0.89	0.64	0.97	0.56	0.97	1.08	0.94	0.86	0.13
Cancer	1.83	1.34	1.15	0.78	1.98	2.61	2.33	2.05	0.65
Vehicle	2.31	1.95	1.32	1.02	2.21	2.89	2.52	2.37	0.85
Car	3.84	3.20	3.32	1.83	3.92	4.61	4.36	4.08	1.54
Abalone	4.15	3.97	4.21	3.42	4.25	5.29	4.88	4.93	2.93
Anneal	1.75	1.63	1.41	1.07	1.83	2.49	2.06	1.97	0.96
Hepatitis	1.61	1.25	1.30	0.76	1.54	2.10	1.95	1.89	0.68

## Data Availability

The data used to support the findings of this study are available from the corresponding author upon request.
